# Bacterial and fungal biomarkers in irritable bowel syndrome (IBS) and inflammatory bowel disease (IBD): trans-kingdom interactions, *Blastocystis* carriage, and enterotype–succinotype stratification

**DOI:** 10.1186/s13099-026-00819-3

**Published:** 2026-04-11

**Authors:** Ayman A. El-Badry, Abdulaziz A. Al-Quorain, Nehal Hosin, Mark van der Giezen, Yohannes Seyoum

**Affiliations:** 1 Department of Microbiology, College of Medicine, Imam Abdulrahman Bin Faisal University, Dammam, Saudi Arabia; 2 Department of Internal Medicine and Gastroenterology, College of Medicine, Imam Abdulrahman Bin Faisal University, Dammam, Saudi Arabia; 3 Department of Chemistry, Bioscience, and Environmental Engineering, University of Stavanger (UiS), Stavanger, Norway; 4 Biosciences University of Exeter, Exeter, UK; 5 Research Department, Stavanger University Hospital, Stavanger, Norway; 6 Natural Resources Institute, University of Greenwich, Chatham Maritime , Kent UK

**Keywords:** Gut microbiome, Irritable bowel syndrome (IBS), Inflammatory bowel disease (IBD), Blastocystis, Enterotype, Succinotype, Bacteria–fungi interactions

## Abstract

The gut microbiome, encompassing bacteria, fungi, and protists, is integral to gastrointestinal (GI) health and disease. While bacterial dysbiosis is well documented in irritable bowel syndrome (IBS) and inflammatory bowel disease (IBD), fungal contributions and bacterial-fungal interactions remain underexplored, particularly in non-Western populations. We profiled bacterial (16S rRNA) and fungal (ITS) communities from stool samples of Saudi participants with IBS, ulcerative colitis (UC), Crohn’s disease (CD), and controls. Analyses incorporated bacterial enterotypes, succinotypes, and *Blastocystis* carriage to explore ecological and metabolic stratification. Differences in community composition were largely explained by GI disorder, host factors, enterotype, and *Blastocystis* carriage, accounting for 1–9% of bacterial and fungal variation. IBS patients exhibited increased bacterial richness and evenness, whereas UC and CD displayed stronger fungal dysbiosis. Enterotyping identified two *Bacteroides*-dominant clusters: Bact1 (with *Faecalibacterium*, *Alistipes*) and Bact2 (enriched in *Escherichia-Shigella*, *Streptococcus*), with Bact2 linked to reduced diversity and elevated calprotectin. Succinotyping distinguished *Dialister*-dominant (D-type) and *Phascolarctobacterium*-dominant (P-type) communities; UC was enriched in fragile, bridge-dependent D-type networks, in contrast to resilient, short-chain fatty acid (SCFA)- and H_2_S-producing P-type networks. *Blastocystis* was detected in 48% of participants (mainly subtypes 1 (ST1) and ST3). It was associated with enrichment of butyrate-producing bacteria (*Roseburia*, *Anaerobutyricum*, *Butyribacter*) and distinct fungal genera, with subtype-specific signatures (ST1 enriched in *Alistipes*, ST3 in *Escherichia-Shigella*). Disease-associated biomarkers included UC-linked depletion of butyrate producers and enrichment of *Cutibacterium*, *Comamonas*, and *Dialister*; depletion of *Clostridia UCG-014* in CD; and enrichment of fermentative fungi (*Candida*, *Meyerozyma*) and bacteria (*Coprococcus*, *Catenibacterium*) in IBS. Cross-domain networks were resilient and modular in controls and IBS but fragile in UC and intermediate in CD. These findings highlight trans-kingdom interactions and *Blastocystis* carriage as ecological features of the gut microbiome in IBS and IBD, with enterotype-succinotype stratification offering complementary biomarkers for disease classification in a non-Western cohort.

**Supplementary Information:**

The online version contains supplementary material available at 10.1186/s13099-026-00819-3.

## Introduction

The gut microbiome, comprising bacteria, fungi, and other microbes, plays a pivotal role in gastrointestinal (GI) health and disease. While bacterial dysbiosis is well-documented in irritable bowel syndrome (IBS) and inflammatory bowel disease (IBD) [1–3], emerging evidence highlights fungi as key contributors to mucosal inflammation, metabolic dysfunction, and symptom severity [4, 5]. Trans-kingdom interactions, particularly bacterial-fungal crosstalk are increasingly recognized as critical regulators of gut ecosystem stability, yet their roles in GI disorders remain poorly characterized [6, 7], especially in non-Western populations.

In IBD, pathogenesis is strongly associated with gut dysbiosis, particularly shifts in *Bacteroides* and *Faecalibacterium*. Bacteroides-dominated communities show disease-associated compositional and functional shifts in IBD, with altered abundance and metabolic activity across disease states [8]. Several Bacteroides species have been implicated in immune modulation in experimental models. Reduced *Faecalibacterium prausnitzii* abundance and butyrate production are associated with impaired intestinal homeostasis, while experimental supplementation ameliorates colitis in animal models [9, 10]. Importantly, *F. prausnitzii* abundance is considered a robust biomarker for disease activity, treatment response, and recurrence in IBD.

In contrast, IBS is more often linked to fermentative taxa such as *Bifidobacterium* and *Lactobacillus*, reflecting distinct microbial signatures across GI disorders [11]. Fungal dysbiosis, particularly in IBD, is characterized by *Candida* overgrowth and Basidiomycota-to-Ascomycota ratios, correlating with disease activity and immune activation [12, 13]. However, most studies focus on single kingdoms, neglecting how bacterial-fungal interactions drive or mitigate pathology. For instance, *Candida* can exacerbate bacterial dysbiosis by degrading mucin, while *Saccharomyces* may suppress inflammation via metabolite cross-feeding [14, 15].

Geographical and ethnic disparities further complicate this picture. Existing microbiome studies disproportionately represent Western cohorts, which constitute only a small fraction of global diversity, despite evidence that diet, genetics, and environmental exposures distinctly shape gut microbial ecology [16, 17]. This underscores the critical need to investigate microbial communities in non-Western populations, particularly in the Middle East, where unique dietary patterns and genetic factors may yield novel insights. However, we note that individual-dietary intake was not recorded in this study. Because diet may differ systematically by disease status (e.g., symptom-driven restriction) as well as by cultural context, it represents an important unmeasured factor when interpreting microbiome-disease association in this cohort. Saudi Arabia, with rising rates of IBS [18, 19], offers an understudied yet important context to explore trans-kingdom interactions. Furthermore, no studies to date have integrated bacterial enterotypes, succinotypes (succinate-driven communities), and fungal networks in these populations, despite their potential to explain region-associated disease phenotypes.

Studies indicate that *Blastocystis* interacts with both the prokaryotic and eukaryotic gut microbiome. Colonization has been associated with higher bacterial diversity and broad restructuring of the gut ecosystem, including shifts in bacterial, fungal, and other eukaryotic taxa [20, 21]. While often reported as a common component of the healthy gut microbiome [22], genetic variation among subtypes suggests that *Blastocystis* cannot be uniformly considered commensal. In particular, subtypes ST4 and ST7 have demonstrated pathogenic potential and pro-inflammatory effects in experimental studies [20]. These findings highlight the complex role of *Blastocystis* within the gastrointestinal ecosystem and underscore the need to investigate its associations with gut disorders, including IBD (UC and CD) and IBS.

To this end, using a multi-biome approach, we analysed bacterial and fungal communities in a Saudi cohort (IBS, UC, CD, healthy controls) to define trans-kingdom interaction networks, identify inflammation/age-stratified biomarkers, and characterize bacterial enterotypes and succinotypes. Intra-kingdom network analysis of succinotypes revealed functional niches and keystone taxa linked to gastrointestinal disorders.

## Materials and methods

### Patient recruitment

Stool samples were collected at the microbiology laboratory of King Fahad hospital, Kingdom of Saudi Arabia. Participants were classified into healthy controls (*n* = 9), irritable bowel syndrome (IBS, *n* = 29), ulcerative colitis (UC, *n* = 31), and Crohn’s disease (CD, *n* = 30). Inclusion criteria encompassed all specimens submitted to the laboratory for routine ova and parasite examination as part of the patient’s management for individuals presenting with gastrointestinal disorders or for routine checkups. Participants who were unable to comply with the study protocol or who declined to provide written informed consent were excluded.

#### Ethics statement

The study protocol was reviewed and approved by the Deanship of Scientific Research, Imam Abdulrahman Bin Faisal University, Dammam, Saudi Arabia (IRB-2021-01-009). All procedures involving human participants were conducted in accordance with the ethical standards of the institutional research committee, the regulations of the Kingdom of Saudi Arabia governing the protection of human research participants, and the Declaration of Helsinki.

#### Informed consent

Written informed consent was obtained from all participants prior to sample collection. All participants provided voluntary consent after being informed of the study’s purpose and procedures.

### DNA extraction

Copro-DNA was extracted from ~ 200 mg of stool using the QIAamp Fast DNA Stool Mini Kit (QIAGEN) with modifications. Approximately 200 mg of 400–600 μm sterile glass beads (Sigma-Aldrich) were added, along with 1 mL of InhibitEX buffer, and the mixture was vortexed continuously until homogenized (for more than 1 min). The suspension was heated at 95 °C for 5–10 min, vortexed for 15 s, and centrifuged at full speed. If particulates remained, the supernatant was re‑centrifuged. Then, 600 µL of the supernatant (avoiding solids) was transferred to a 2 mL tube with 25 µL Proteinase K and 600 µL Buffer AL, vortexed for 15 s, and incubated at 56 °C overnight. The manufacturer’s protocol was followed thereafter.

### *Blastocystis* detection

*Blastocystis* DNA was amplified from extracted copro-DNA using primers (The RD5: 5′-ATCTGGTTGATCCTG CCAGT-3′ and BhRDr: 5′-GAGCTTTTTAACTGC AACAACG-3′) targeting *Blastocystis* species-specific SSU rDNA fragment of 550–585 bp [23]. PCR reaction and conditions were done according to Scicluna et al. (2006) [23]. Amplified PCR products were purified, and Sanger sequencing was performed using primers RD5 and BhRDr in both directions. The resulting sequences were aligned with reference sequences in GenBank using the BLAST algorithm (National Center for Biotechnology Information, NCBI: https://blast.ncbi.nlm.nih.gov/Blast.cgi) to determine *Blastocystis* subtypes.

### Sequencing and bioinformatics analysis

Faecal DNA from 101 participants was sequenced using Illumina NovaSeq 6000 in paired-end mode (2 × 250 bp). The V3-V4 region of the 16 S rRNA gene was amplified using the primers 341 F (CCTAYGGGRBGCASCAG) and 806R (GGACTACNNGGGTATCTAAT). The internal transcribed spacer (ITS) region was amplified using ITS1 primer ITS5-1737 F (GGAAGTAAAAGTCGTAACAAGG), and ITS2-2043R (GCTGCGTTCTTCATCGATGC). Primers were removed using Cutadapt [24].

Both 16S and ITS sequences were processed using DADA2 (v1.32.0) [25]. Forward and reverse reads were truncated at 227 and 225 bp, respectively with maxN = 0 and maxEE = 2. ITS sequences were processed with a minimum post-trimming length of 50 bp. ASVs were inferred in “pool” mode, paired-end reads merged, and chimeras removed using the consensus method. 16S ASVs were taxonomically classified using the q2-feature-classifer with a naïve Bayes approach using SILVA database (version 138.2) [26]. The SILVA reference sequences, and taxonomy files were pre-formatted with RESCRIPt [27]. A primer-specific (341F and 806R) feature classifier was generated from this database to improve classification accuracy. ITS ASVs were taxonomically assigned using DADA2’s assign taxonomy function using RDP classifier against UNITE database, version 10 [28]. Singleton and rare ASVs with a relative abundance below 0.1% of the mean sequencing depth were removed from both the 16S and ITS datasets. Furthermore, chloroplast and mitochondria sequences were removed from 16S data. The 16S rRNA dataset contained14,684,352 reads (median = 161,208 reads/sample; 11 phyla, 242 genera), and the ITS dataset contained 5,309,084 reads (median = 57,286 reads/sample; 12 phyla, 431 genera).

Rarefaction depths were set at 77,258 reads/sample for 16S (supplementary Fig. 1A) and 36,560 reads/sample for ITS (supplementary Fig. 1B); samples with insufficient depth were excluded. Alpha diversity metrics were calculated with phyloseq [29], testing normality using the Shapiro-Wilk test. Parametric (t-test) or non-parametric (Wilcoxon signed-rank) tests were applied as appropriate. Pearson or Spearman correlations were used for continuous associations. Beta diversity was evaluated using Bray-Curtis and Jaccard distances, with group differences tested via PERMANOVA (vegan R package) [30] and pairwise comparisons (pairwiseAdonis) [31]. The variance explained by each factor (R² × 100%) was reported, with marginal tests adjusting for GIT category.

#### Gut enterotype, succinotype classification and fungal dysbiosis

Gut enterotypes were inferred from rarefied genus-level 16S counts using Dirichlet multinomial mixtures (DMM) as described by Holmes et al. (2012) [32]. Two clusters were optimal based on Bayesian Information Criterion (BIC), the Akaike Information Criterion (AIC), and Laplace approximation (Supplementary Fig. 2). Contributions of individual genera to cluster classification were determined from mean absolute differences between multi-component and single-component models. Associations between enterotype classification or GIT disorder group and categorical variables were evaluated using Fisher’s exact tests, with P-values adjusted for multiple comparisons using the Benjamini-Hochberg (BH) procedure. Associations with age were assessed using the Wilcoxon signed-rank test, with statistical significance defined as *P* < 0.05.

Succinotype classification was performed based on dominance of succinate-utilizing genera *Phascolarctobacterium* (P-type) or *Dialister* (D-type), following O’Sullivan et al., (2025) and Anthamatten et al., (2024).

ASVs assigned to *Dialister* (*n* = 23) and *Phascolarctobacterium* (*n* = 3) were aggregated by summing read counts per genus. For each sample, the relative abundance of *Dialister* was calculated as: $\:rD=nD/(nD+nP)$ where $\:nD$ and $\:nP$ represents the read counts of *Dialister* and *Phascolarctobacterium*, respectively. Samples were classified as D-type if $\:rD>0.9$ (*Dialister* was at least 10 times more abundant than *Phascolarctobacterium*) and as P-type if $\:rD<0.1$, and excluded if total reads from both genera were < 10. For individuals with multiple samples, clear classifications (i.e., D-type or P-type) were prioritized over mixed cases. Associations with GIT disorders were assessed using Fisher’s exact test (*P* < 0.05). Furthermore, the Basidiomycota/Ascomycota ratio, indicator of fungal dysbiosis [2], was calculated from ITS phylum-level counts and log-transformed for visualization.

#### Differential abundance analysis

Genera present in < 10% of samples were excluded from both datasets. Associations with metadata were assessed using Wilcoxon rank-sum tests and Analysis of Composition of Microbiomes with Bias Correction 2 (ANCOM-BC2) [33], adjusting for covariates (continuous variables (age) converted to tertiles) and controlling FDR with BH correction (*P* < 0.05).

#### Network inference and analysis

Microbial association networks were inferred using the Sparse InversE Covariance estimation for Ecological Association and Statistical Inference (SPIEC-EASI) pipeline [34]. We constructed intra‑domain bacterial-bacterial and cross-domain bacterial-fungal networks separately by group (controls, IBS, UC, CD) and by succinotype, respectively. Genera present in < 20% of samples were filtered prior to analysis.

Networks were inferred using the sparse graphical lasso (glasso) with Stability Approach to Regularization Selection (StARS) [35] applied to centred log-ratio (CLR) transformed data. StARS performs repeated subsampling of the data (80% subsampling; 50 repetitions) and selects the regularization parameter that maximizes edge stability under perturbation, thereby favouring reproducible network structures and mitigating overfitting in high-dimensional settings.

Isolated vertices (degree < 1) were removed, and networks were converted to *igraph* objects [36]. Community detection was performed using the Leiden algorithm (resolution = 0.5) incorporating both positive and negative edges. Network-level metrics included modularity, global transitivity, edge density, average path length, and diameter. Node-level properties included degree, betweenness, closeness, eigen-centrality, hub scores, and local transitivity. Keystone taxa were identified as the top 5% of nodes by combined z-scored centrality measures (degree, betweenness, closeness, eigen-centrality) [37]. Because SPIEC-EASI infers microbial association structure solely from abundance data, inferred edges represent conditional dependencies among taxa and do not explicitly adjust for host or environmental covariates such as age and disease in case of succinotype network analysis. For visualization, small communities (≤ 2 nodes) were removed, and networks were visualized using Gephi [38] with the Fruchterman-Reingold layout [39].

## Results

### Study characteristics

We analysed 99 participants: 9 controls, 29 IBS, 31 UC, and 30 CD (supplementary Table 1). Age differed significantly across groups (Kruskal-Wallis test, *P* < 0.05), with CD patients being youngest (median = 24 years vs. IBS: 36 years; controls: 41 years). Abdominal pain (79%) and nausea (38%) were most prevalent in IBS (*P* < 0.05, Fisher’s exact test), while vomiting was elevated in controls (44%, *P* < 0.05). Given this age imbalance, we adjusted or stratified analyses accordingly in later diversity and differential abundance tests. Calprotectin positivity (indicating active inflammation) was higher in UC (72%) and CD (60%). *Blastocystis* prevalence was highest in controls (78% vs. 37–55% in disease groups), with subtype 1 (ST1) predominating. Sequencing yielded 2,255 bacterial ASVs (242 genera) and 1,212 fungal ASVs (431 genera).

### Interindividual variation and diversity of bacterial and fungal communities

**Fig. 1:**
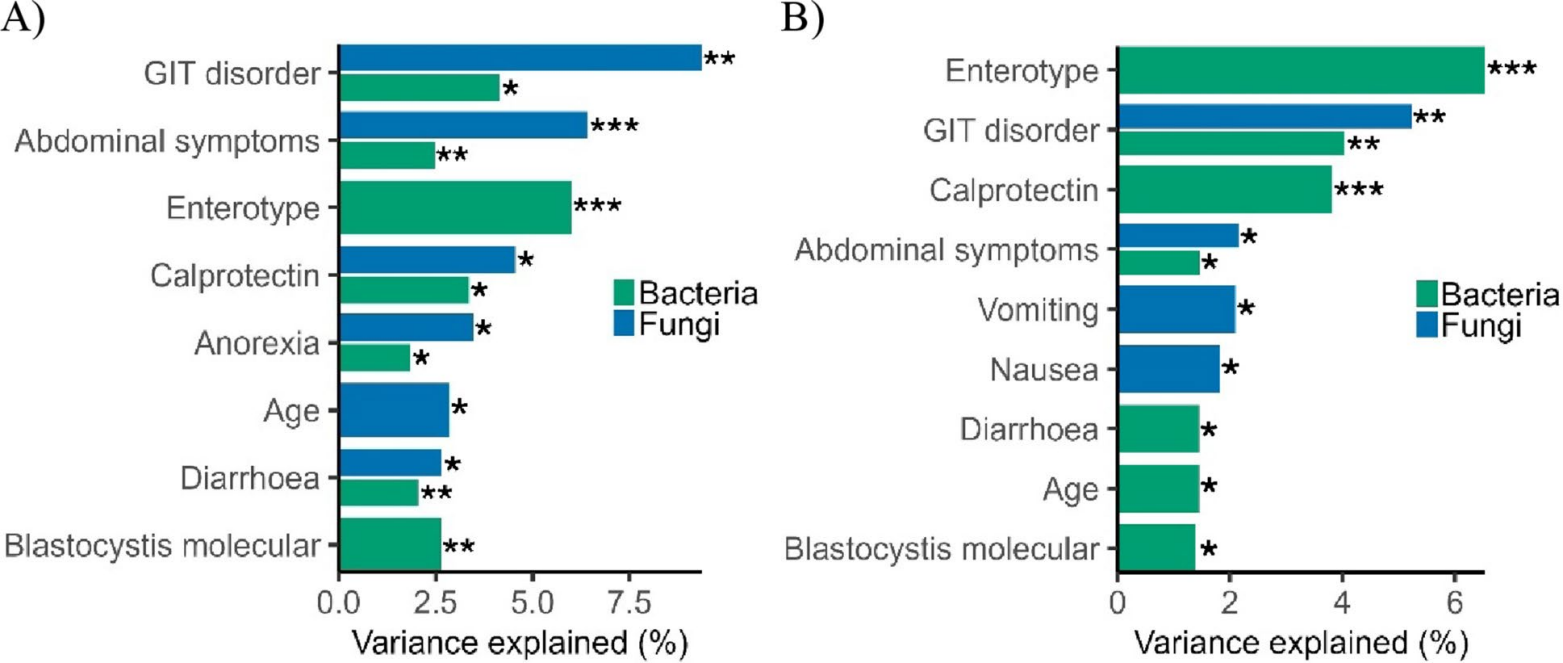
Host and microbial factors shaping gut bacterial and fungal community structure. Variance explained (R² × 100) from PERMANOVA analyses of beta diversity, representing the proportion of interindividual variation in microbial community structure attributed to each factor. Beta diversity was calculated using (**A**) Bray-Curtis dissimilarity at the genus level, (**B**) Jaccard distance. Only factors with statistically significant effects (PERMANOVA, *P* < 0.05) are shown, ordered by effect size. Significance levels: *P < 0.05, **P < 0.01, ***P < 0.001

PERMANOVA indicated that microbiome-related factors collectively explained 1.4–6.5% of variation in bacterial composition (genus-level Bray-Curtis and ASV-level Jaccard, Fig. 1A-B). Several associations persisted after controlling for GI disorder (see below), indicating that both disease status and other host factors independently shape microbial structure. Enterotype was the strongest bacterial predictor (~ 6.5%; *P* = 0.001). *Blastocystis* presence explained 1.4% (Jaccard, *P* = 0.03) and 2.6% (Bray-Curtis, *P* = 0.003) of bacterial variation; ST1 vs. ST3 did not differ significantly. No microbiome‑related factors were significantly associated with fungal diversity.

Host‑associated factors explained 1.4–4.2% of bacterial variation, while fungal communities showed stronger host structuring (1.8–9.4%; *P* < 0.05, Fig. 1A-B). GI disorder status explained ~ 4% of bacterial and 5.2–9.4% of fungal variation (*P* < 0.05). Pairwise Bray-Curtis comparisons indicated CD differed from IBS (bacteria *P* = 0.005; fungi *P* = 0.004) and from UC (fungi *P* = 0.008); IBS differed from UC (bacteria *P* = 0.03; fungi *P* = 0.02).

Additional host factors were associated with bacterial composition: calprotectin (Jaccard 3.8%, *P* = 0.001; Bray-Curtis 3.4%, *P* = 0.016), abdominal symptoms (2.5%, *P* = 0.002), diarrhoea (2.1%, *P* = 0.006), anorexia (1.8%, *P* = 0.028), and age (1.4%, *P* = 0.01). In fungi, abdominal symptoms (6.4%, *P* = 0.001), calprotectin (4.5%, *P* = 0.02), anorexia (3.5%, *P* = 0.01), age (2.9%, *P* = 0.02), and diarrhoea (2.6%, *P* = 0.03) were significant. Although significant, these effects explained only a small proportion of variation (R² 1–6%), consistent with modest but consistent shifts in community structure.

When controlling for GI disorder, enterotype remained strongly associated with bacterial composition (5.7–6.3%, *P* < 0.001), with independent effects of calprotectin (3.2–3.4%, *P* = 0.001–0.02), diarrhoea (1.5–2.0%, *P* = 0.01–0.02), anorexia (1.7%, *P* = 0.03), and *Blastocystis* presence (1.4–1.8%, *P* = 0.02–0.03). In fungal communities, abdominal symptoms explained 3.4% of variation (Bray-Curtis; *P* = 0.01) after controlling for GI disorder.

**Fig. 2:**
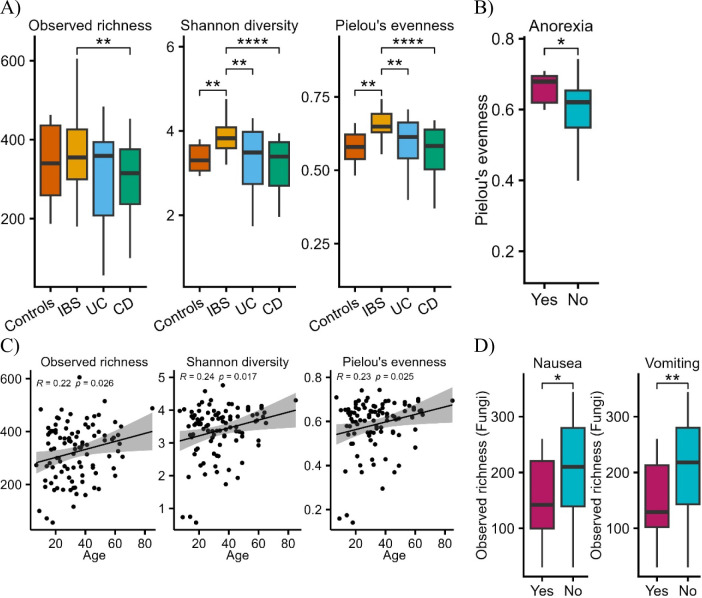
Alpha diversity of bacterial and fungal communities across gastrointestinal disorders and host factors. (**A**) Comparisons of observed richness, Shannon diversity, and Pielou’s evenness across GI disorder groups (controls, IBS, UC, CD). (**B**) Pielou’s evenness between individuals with and without anorexia. (**C**) Pearson correlations between age and alpha diversity metrics, with correlation coefficients (r) and *P*-values indicated. (**D**) Comparisons of observed richness in the fungal community between individuals with and without vomiting and nausea. Statistical significance for categorical comparisons was assessed using the Wilcoxon rank-sum test (*P < 0.05, **P < 0.01, ***P < 0.001). IBS, irritable bowel syndrome; UC, ulcerative colitis; CD, Crohn’s disease

Bacterial alpha diversity (observed richness, Shannon, Pielou’s) was higher in IBS compared to other groups: richness greater than CD (Wilcoxon rank‑sum, *P* = 0.02); evenness higher in IBS vs. controls (*P* = 0.01), UC (*P* = 0.03), and CD (*P* < 0.001); Shannon elevated in IBS vs. all groups (*P* < 0.05, Fig. 2A). Anorexia was associated with increased evenness (Fig. 2B). Age correlated positively with richness (Pearson *r* = 0.23, *P* = 0.02), Shannon (*r* = 0.24, *P* = 0.02), and evenness (*r* = 0.20, *P* = 0.02, Fig. 2C).

Fungal alpha diversity differed by vomiting and nausea: individuals without vomiting or nausea exhibited higher observed richness (Wilcoxon rank‑sum, *P* < 0.05, Fig. 2D), suggesting suppression of fungal complexity by these symptoms.

### Gut microbial enterotype

**Fig. 3:**
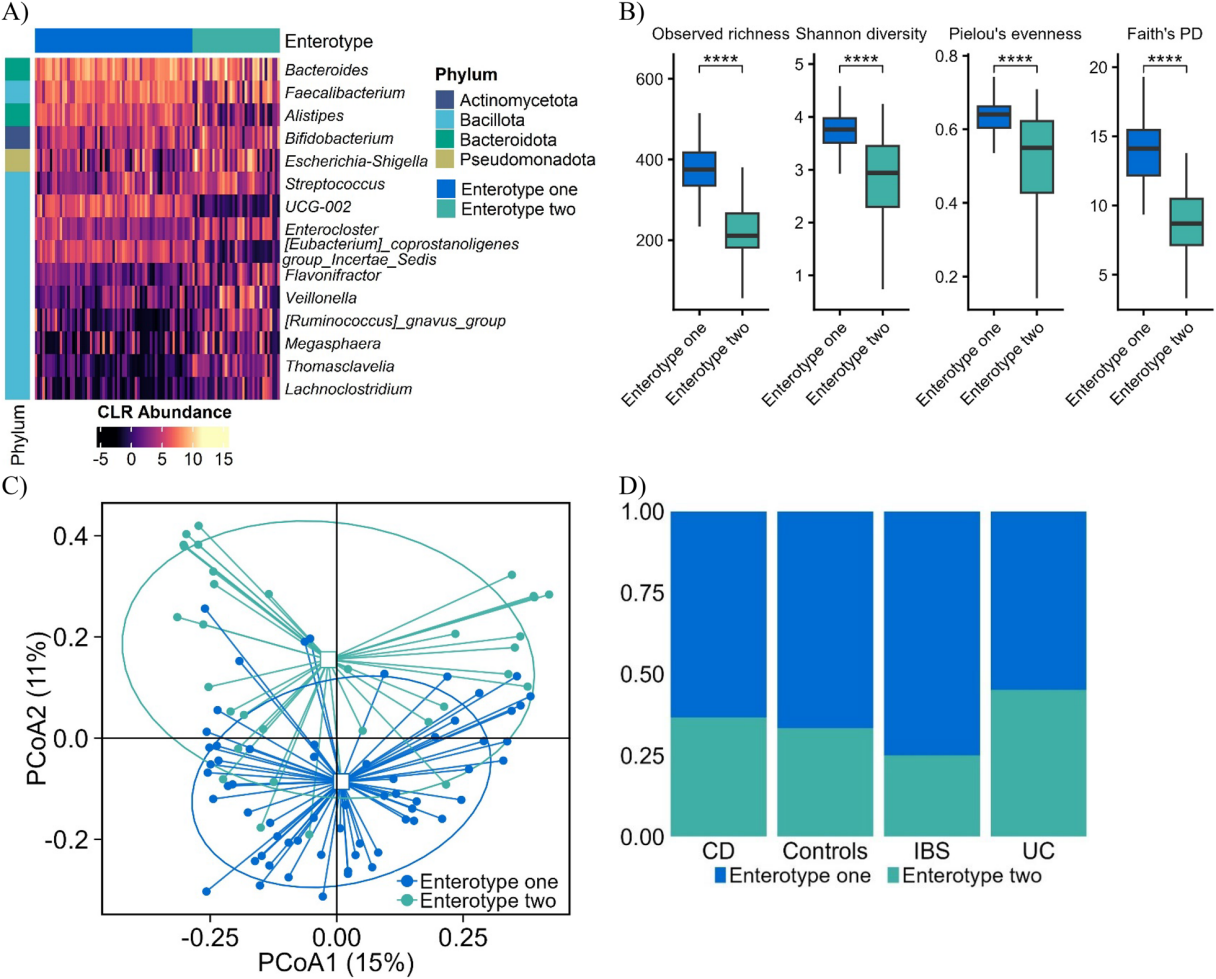
Enterotype Classification Based on Dirichlet Multinomial Modelling (DMM). (**A**) Heatmap depicting the relative abundance of the top 15 bacterial genera contributing most to enterotype differentiation. Contributions were determined by calculating the sum of mean absolute differences in centred log-ratio (CLR) transformed data between DMM models with two components (k = 2) versus a single component (k = 1). (**B**) Box plots representing alpha diversity metrics, including observed richness, Shannon diversity, and Pielou’s evenness. (**C**) Principal Coordinates Analysis (PCoA) plot based on Bray-Curtis dissimilarity at the genus level, demonstrating significant differences between enterotype groups (*P* = 0.001). (**D**) Bar plot illustrating the proportion of the two identified enterotypes across different groups: irritable bowel syndrome (IBS), ulcerative colitis (UC), Crohn’s disease (CD), and healthy controls. Statistical significance was assessed using the Wilcoxon rank-sum test (*P < 0.05, **P < 0.01, **P < 0.001)

DMM modelling identified two enterotypes using BIC/AIC/Laplace. Both were *Bacteroides*-dominant; Bact1 also harboured *Faecalibacterium* and *Alistipes*, whereas Bact2 was enriched in *Escherichia-Shigella* and *Streptococcus* (Fig. 3A). Bact2 exhibited reduced alpha diversity (Fig. 3B) and separated in Bray-Curtis PCoA (*P* = 0.001, Fig. 3C). This enterotype shares similarities with previously described enterotypes associated with obesity [40], inflammatory bowel disease (IBD), and primary sclerosing cholangitis [40, 41]. Furthermore, previous work has linked similar enterotypes to looser stools and elevated levels of intestinal and systemic inflammation markers [41]. These findings suggest that Bact2 may represent a gut microbial community state indicative of dysbiosis and inflammatory conditions. Enterotype distribution itself did not differ across disease groups, but inflammatory potential (calprotectin positivity) was higher in Bact2 individuals.

However, in our study, enterotype distribution was similar across the control and disease groups (IBS, UC, and CD) as indicated by Fisher’s exact test (Fig. 3D). While enterotype distribution did not differ between disease groups, Bact2 individuals exhibited higher calprotectin positivity (81% vs. 54%, BH *P* = 0.02), indicating that enterotype may modulate inflammatory potential rather than classify disease.

### Succinotype classification and interaction with host-associated factors

**Fig. 4:**
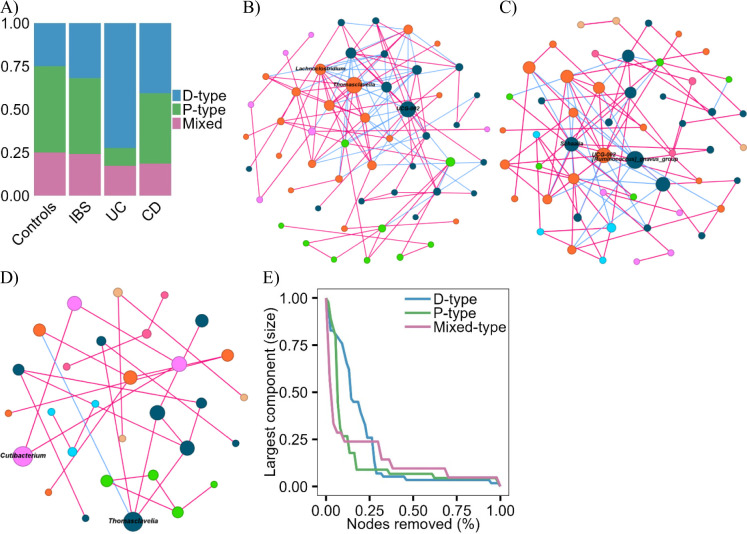
Succinotype distribution and network properties. (**A**) Prevalence (%) of succinotypes across groups. (**B**-**D**) Co-occurrence networks inferred with Sparse and Compositionally Robust Inference of Microbial Ecological (SPIEC-EASI) for D-type (**B**), P-type (**C**), and mixed-type (**D**, **E**) Network robustness curves. Nodes represent bacterial genera, sized by combined keystone score, and labelled for keystone taxa. Edges represent co-occurrence relationships (pink = positive, blue = negative)

Across all groups, 43% of participants were D-type, 30% P-type, 18% mixed-type, and 9% were unclassified due to low reads. In UC, D-type prevalence was higher (24%) than in controls (2%, BH *P* = 0.04) and IBS (9%, BH *P* = 0.03), whereas P-type prevalence was lower (3%) than in IBS (12%, BH *P* = 0.03), and mixed-type prevalence was elevated (6%) relative to controls (2%, BH *P* = 0.04) (Fig. 4A). Thus, UC was specifically enriched in D-type communities, consistent with a link between succinate metabolism and disease-associated instability. As shown in subsequent network analyses, these D-type communities also exhibited fragile, bridge-dependent connectivity, consistent with UC-associated instability.

Differential abundance analysis revealed succinotype-specific microbial signatures (supplementary Table 2). *Bacteroides* was enriched in P-type (26.1%) compared to D-type (17.5%, *P* = 0.03) and mixed-type (12.3%, *P* = 0.02). *Bilophila*, a hydrogen sulphide producer, was significantly higher in P-type than D-type (*P* = 3.3 × 10⁻⁴). Members of the *Eubacterium coprostanoligenes* and *Eubacterium eligens groups*, both linked to cholesterol and polysaccharide metabolism, were enriched in mixed- and D-type communities. *Butyribacter* and *Christensenellaceae R-7 group*, both short-chain fatty acid (SCFA) producers, were more abundant in P-type, whereas *Lachnoclostridium* and *Enterocloster* predominated in D-type.

Additional associations included enrichment of *Clostridia UCG-014* and *Faecalicatena* in P-type, while *Coprobacter* and *Methylorubrum* were more frequent in mixed and D-type samples. *Streptomyces* and *Turicimonas* were differentially abundant in mixed-type communities. Collectively, these patterns highlight functional shifts across succinotypes: P-type communities favour hydrogen sulphide and SCFA production, while D-type communities are enriched for taxa lined to polysaccharide degradation and alternative fermentation pathways, aligning with UC-associated metabolic disruption.

To explore ecological interactions among bacteria, co-occurrence networks were inferred from 16S rRNA profiles using SPIEC-EASI. D-type bacterial networks displayed a fragile architecture (transitivity = 0.38, path length = 172.6, diameter = 1951.3, density = 0.048, modularity = 0.36, 138 edges: 96 positive, 42 negative), with keystone taxa such as *Thomasclavelia*, *UCG-002*, and *Lachnoclostridium* acting as critical bridges (Fig. 4B). Sequential removal of nodes with high betweenness centrality confirmed this fragility: elimination of 18.5% of nodes reduced the largest connected component to 50% of its original size, and removal of 37% reduced it to 12.9% (Fig. 4E). This vulnerability parallels the instability observed in UC-specific networks overall.

In contrast, P-type networks were more resilient and tightly clustered (transitivity = 0.28, path length = 97.9, diameter = 519.0, density = 0.038, modularity = 0.43, 103 edges: 74 positive, 29 negative), with keystone taxa such as *Schaalia* and *UCG-002* supporting stable connectivity (Fig. 4C). Mixed-type networks were sparse (41 edges: 30 positive, 11 negative) but displayed a high proportion of positive interactions, suggesting ecological flexibility that may facilitate transition toward P-type stability (Fig. 4D).

Taken together, D-type enrichment in UC, coupled with its fragile, bridge-dependent network structure, reinforces a mechanistic link between succinate-driven communities and UC-associated instability. By contrast, P-type networks reflect ecological resilience with minimal disease association. Conceptually, enterotypes capture broad community structure, while succinotypes resolve metabolic potential and interaction networks. This functional complementarity provides an additional ecological layer to interpret microbial signatures in GI disorders.

### *Blastocystis* prevalence and associations

*Blastocystis* was detected in 48% of participants, with subtypes (ST) 1 and ST3 accounting for 77% and 22% of positive cases, respectively. While *Blastocystis* status modestly associated with overall bacterial diversity, it did not associate with clinical variables, enterotype and succinotypes (Fisher’s Exact Test, *P* > 0.05). Nonetheless, *Blastocystis* carriage was linked to distinct bacterial and fungal signatures, prompting further exploration of taxonomic differences (supplementary Table 3).

Given the association between *Blastocystis* carriage and bacterial beta diversity, we examined genus-level differences using Wilcoxon rank-sum tests. Thirteen bacterial genera showed significant differences between *Blastocystis*-positive and -negative individuals (BH *P* < 0.05), nine of which belonged to Bacillota. *Bacteroides* was more abundant in *Blastocystis*-negative individuals (24.4% vs. 12.8%; *P* = 0.012). In contrast, *Blastocystis*-positive participants were enriched in several butyrate-producing taxa, including *Roseburia* (1.5% vs. 0.75%; *P* = 0.019), *Anaerobutyricum* (0.35% vs. 0.07%; *P* = 0.003), and *Butyribacter* (0.11% vs. 0.06%; *P* = 0.041), as well as *Phascolarctobacterium* (2.5% vs. 1.4%; *P* = 0.044). Additionally, differentially abundant genera included *Anaerococcus*, *Paludicola*, *Neisseria*, *Peptostreptococcus*, *Mitsuokella*, *Fusicatenibacter*, and *Leyella* (*P* < 0.05).

Subtype-specific comparisons revealed eight bacterial genera that differed between ST1 and ST3 carriers. ST1 was associated with enrichment of *Alistipes*, *Terrisporobacter*, *Agathobacter*, and *Anaeroglobus*, whereas ST3 carriers harboured higher levels of *Escherichia-Shigella*, *Klebsiella*, *Fusobacterium*, and *Eikenella*.

*Blastocystis* carriage was also linked to fungal community variation. Six genera showed significant differences between *Blastocystis*-positive and -negative individuals (BH *P* < 0.05). *Blastocystis*-positive participants were enriched in *Nigrospora*, *Capronia*, *Naganishia*, *Alternaria*, and *Lunulospora*.

Subtype comparisons identified six additional fungal taxa: ST1 was enriched in *Marquandomyces*, *Zygosporium*, unclassified genera within Sclerotiniaceae, and Sporormiaceae, while ST3 carriers showed higher levels of *Didymella*.

Together, these results indicate that while *Blastocystis* prevalence and subtype distribution were not associated with host characteristics, their presence corresponded to distinct ecological signatures. *Blastocystis* carriage was linked to enrichment of butyrate-producing bacteria and diverse fungal taxa, whereas subtype-specific differences highlighted contrasting associations with commensal versus potentially pathogenic genera.

### Taxonomic composition and biomarkers across GIT groups

**Fig. 5:**
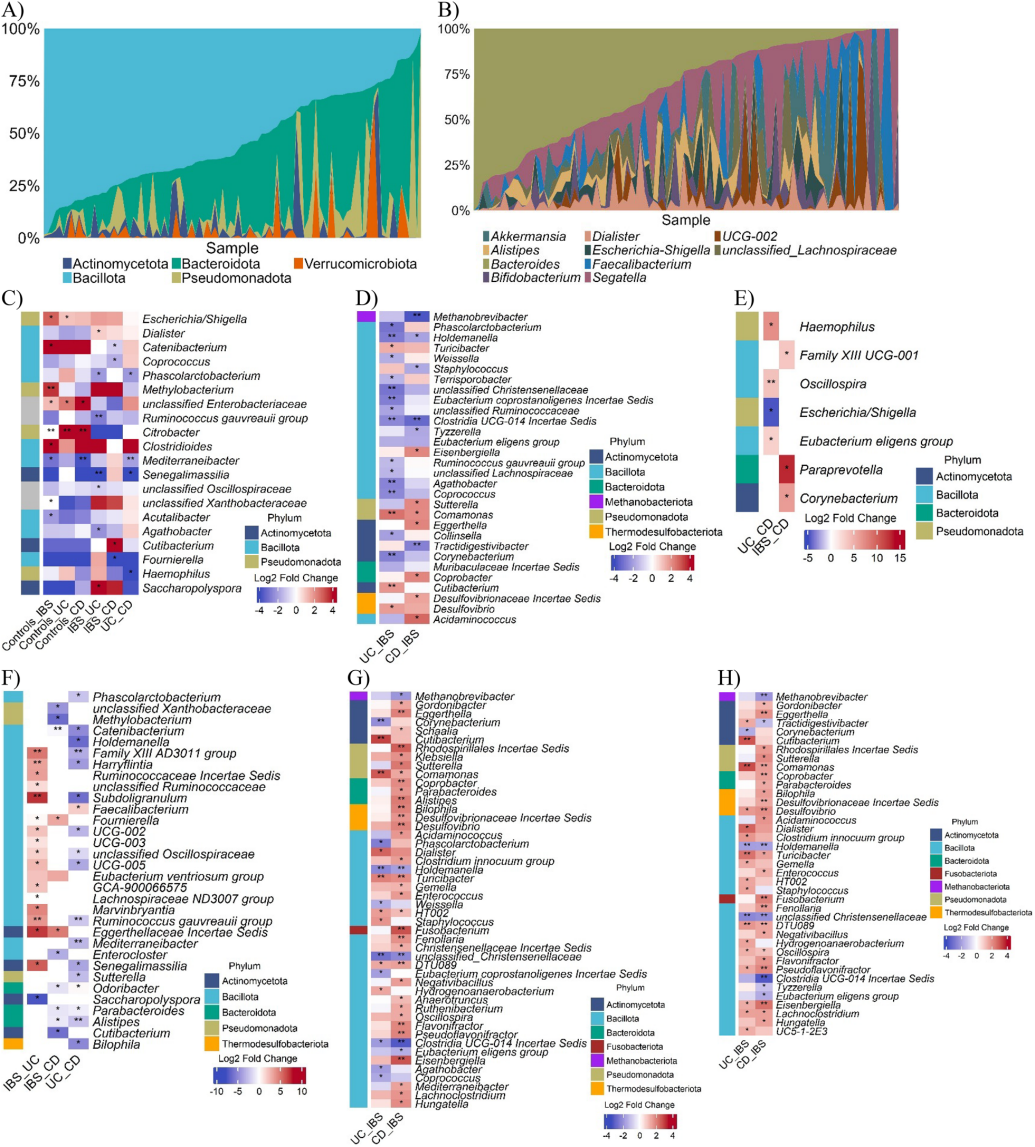
Relative abundance (%) and differential abundance of gut microbiota across gastrointestinal disorders. This figure combines an area plot and multi-panel heatmaps to illustrate the microbial composition and differential abundance in healthy controls, irritable bowel syndrome (IBS), ulcerative colitis (UC), and Crohn’s disease (CD). (Top Panel) Area plot showing the relative abundance (%) of the top bacterial and archaeal taxa in the study population, with each bar representing an individual sample. (Bottom Panels) Multi-panel heatmap displaying raw log-fold changes (LFC) for genera with significant differential abundance across groups. Each panel represents a distinct analysis, with rows indicating genera and columns representing pairwise group comparisons. The colour gradient indicates LFC (blue: depletion in group 1 vs. group 2; red: enrichment in group 1 vs. group 2). Significance is denoted by asterisks (*Benjamini-Hochberg (BH) adjusted *P*-value < 0.05, **BH adjusted *P*-value < 0.01). Genera are coloured by phylum. (**A**) Top five phyla in the full cohort. (**B**) Top 10 genera in the full cohort. (**C**) Wilcoxon rank-sum test on across GIT groups. (**D**) ANCOM-BC2 across GIT groups after adjusting for age (tertile) in disease groups. (**E**) Wilcoxon rank-sum test in individuals without abdominal symptoms (see the results for criteria used) in disease groups. (**F**) Wilcoxon rank-sum test in individuals with abdominal symptoms. (**G**) ANCOM-BC2 across GIT groups after adjusting for gut enterotypes in disease groups (H). ANCOM-BC2 across GIT groups after adjusting for *Blastocystis* (positive/negative) groups

Bacterial communities were dominated by Bacillota (52%), Bacteroidota (29%), Pseudomonadota (11%), and Actinomycetota (4%) (Fig. 5A). At genus level, *Bacteroides* (34%), *Faecalibacterium* (19%), *Escherichia/Shigella* (8%), *Akkermansia* (7%), and *Alistipes*, *Dialister*, *Segatella*, and *Bifidobacterium* (5–6% each) were most abundant (Fig. 5B).

Univariate analyses (Wilcoxon, BH *P* < 0.05) identified 16 genera differing across groups (Fig. 5C). Notable patterns included higher levels of the fermentative taxon *Dialister* in UC, and IBS-specific enrichment of SFCA-producing commensals such as *Catenibacterium* and *Coprococcus*. The propionate producer *Phascolarctobacterium* varied across groups, with higher levels in CD than UC and IBS. Low-abundance taxa also distinguished groups, including the fibre-fermenting *Segatella*, and the methylotrophic proteobacterium *Methylobacterium*.

Age-adjusted ANCOM-BC2 revealed broader shifts (30 genera, Fig. 5D). However, many of these associations attenuated once we accounted for inflammatory status (calprotectin), underscoring the influence of inflammation on microbial signatures. UC was characterized by depletion of SCFA-producing commensals (*Phascolarctobacterium*, *Coprococcus*, *Agathobacter*, *Ruminococcus gauvreauii* group) and enrichment of potential opportunists or pro-inflammatory taxa (*Comamonas*, *Cutibacterium*, *Desulfovibrio*, and *Turicibacter*). CD vs. IBS comparisons showed depletion of the methanogen *Methanobrevibacter*, the beneficial commensal *Holdemanella*, and *Clostridia UCG-014*, alongside enrichment of the opportunist *Comamonas*. Several CD-specific losses (*Eggerthella*, *Eisenbergiella*, *Sutterella*) were also noted.

Adjustment for calprotectin reduced significant findings to a single robust marker: strong depletion of *Clostridia UCG-014* in CD, suggesting that many UC- and IBS-associated changes are secondary to inflammation rather than primary disease effects.

Symptom stratification (abdominal pain, bloating, urgency; 2–7% variance in beta diversity) identified 32 genera distinguishing symptomatic patients (Fig. 5E-F). Trends largely mirrored cohort-level results: UC showed reduced levels of SCFA producers such as *Phascolarctobacterium* and *Ruminococcus gauvreauii group*, CD showed enrichment of dysbiosis-associated taxa like *Alistipes* and *UCG-002*, and IBS showed higher *Coprococcus* and *Catenibacterium*. No genera overlapped between symptomatic and asymptomatic subsets, underscoring the symptom-specific nature of microbial alterations.

Microbiome-related covariates further refined associations. After enterotype adjustment, 17 genera (UC vs. IBS) and 37 genera (CD vs. IBS) were significant (Fig. 5G). UC was enriched in potential opportunists (*Cutibacterium*, *Comamonas*) and the fermentative *Dialister*, while depleted in beneficial commensals such as *Christensenellaceae*, *Holdemanella*, *Coprococcus*, and *Phascolarctobacterium*. CD vs. IBS confirmed depletion of the commensal *Clostridia UCG-014* and enrichment of pro-inflammatory or dysbiosis-associated taxa (*Eisenbergiella*, *Fusobacterium*, *Alistipes*, and *Sutterella*). Adjustment for *Blastocystis* yielded similar patterns (Fig. 5H), with *Cutibacterium*, *Comamonas*, and *Dialister* enriched in UC, and *Clostridia UCG-014* depleted in CD. Shared disease-associated genera included Christensenellaceae, *Holdemanella*, *Eisenbergiella*, *Comamonas*, and *Sutterella*.

### Fungal composition and biomarkers across GIT groups

**Fig. 6:**
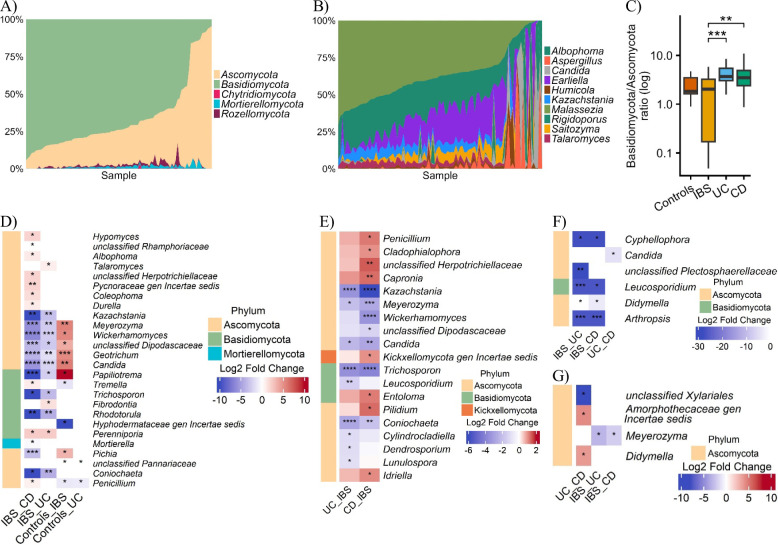
Relative abundance (%) and differential abundance of fungi across gastrointestinal disorders. This figure combines an area plot and multi-panel heatmaps to illustrate the microbial composition and differential abundance in healthy controls, irritable bowel syndrome (IBS), ulcerative colitis (UC), and Crohn’s disease (CD). (Top Panel) Area plot showing the relative abundance (%) of the top fungal taxa in the study population, with each bar representing an individual sample. (Bottom Panels) Multi-panel heatmap displaying raw log-fold changes (LFC) for genera with significant differential abundance across groups. Each panel represents a distinct analysis, with rows indicating genera and columns representing pairwise group comparisons. The colour gradient indicates LFC (blue: depletion in group 1 vs. group 2; red: enrichment in group 1 vs. group 2). Significance is denoted by asterisks (*Benjamini-Hochberg (BH) adjusted P-value < 0.05, **BH adjusted P-value < 0.01). Genera are coloured by phylum. (**A**) Top five phyla in the full cohort. (**B**). Top 10 genera in the full cohort. (**C**). Basidiomycota/Ascomycota relative abundance ratio (Wilcoxon pairwise test). (**D**) Wilcoxon rank-sum test on across GIT groups. (**E**) ANCOM-BC2 across GIT groups after adjusting for age (tertile) in disease groups. (**F**). Wilcoxon rank-sum test in calprotectin positive individuals in disease groups (**G**) Wilcoxon rank-sum test in calprotectin negative individuals in disease groups

Fungal communities were dominated by Basidiomycota (66%) and Ascomycota (32%) (Fig. 6A). *Rigidoporus* (36%), *Malassezia* (20%), and *Saitozyma* (16%) were most abundant (Fig. 6B). The Basidiomycota/Ascomycota ratio was significantly higher in UC and CD compared with IBS, suggesting stronger dysbiosis in IBD (Fig. 6C). As with bacteria, these fungal patterns were further shaped by inflammatory status, as calprotectin stratification later confirmed.

Wilcoxon tests identified 26 differentially abundant genera (Fig. 6D). IBS was enriched in fermentative taxa (*Candida*, *Meyerozyma*, *Wickerhamomyces*), whereas UC/CD showed depletion of *Papiliotrema* and enrichment of opportunists such as *Capronia* and *Entoloma*. Age-adjusted ANCOM-BC2 confirmed these patterns, highlighting severe depletion of *Meyerozyma* and *Kazachstania* in IBD and CD-specific enrichment of Herpotrichiellaceae (Fig. 6E).

Calprotectin stratification refined these trends. Inflammation-positive samples showed reduced diversity with selective enrichment (e.g., *Candida* in UC vs. CD) and depletion of *Didymella* in CD vs. IBS, while calprotectin-negative samples confirmed persistent IBS enrichment of *Meyerozyma* and revealed UC-specific reduction of *Didymella* (Fig. 6F-G). Collectively, fermentative fungi (*Candida*, *Meyerozyma*) marked IBS in non-inflammatory states, whereas *Didymella* emerged as a candidate UC-specific biomarker independent of inflammation.

### Cross-domain interactions across the GIT disorder

**Fig. 7:**
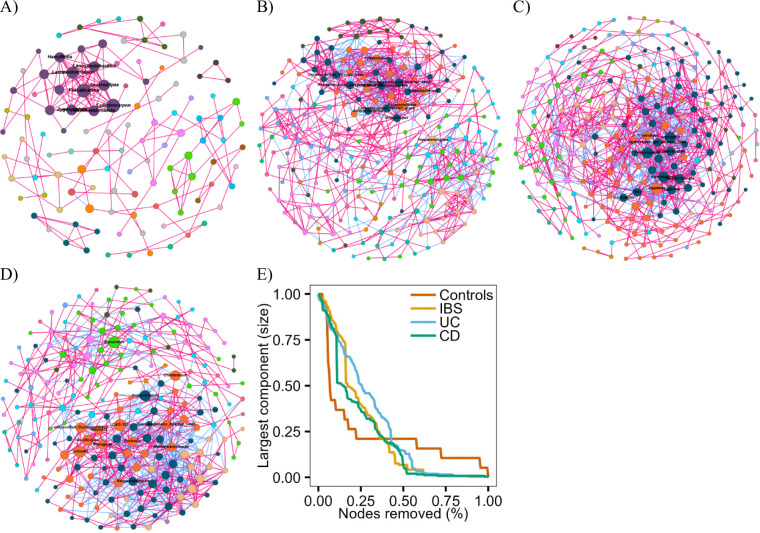
Co-occurrence network inferred by SPIEC-EASI. (**A**) controls, (**B**) IBS, (**C**) UC, (**D**) CD and (**E**) network robustness curve. Network inference with nodes representing taxa at the genus level and edges represents relationships between the genera. Nodes represent bacterial/fungal genera, sized by combined keystone score, and labelled for keystone taxa. Edges represent co-occurrence relationships (pink = positive, blue = negative)

To integrate bacterial and fungal findings, we inferred cross-domain association networks using SPIEC-EASI, revealing marked restructuring of bacterial–fungal associations across GI disorders (Fig. 7). The control network was highly modular and clustered, with keystone taxa including fungal *Cylindrocarpon* and *Zygosporium* and bacterial butyrate producers such as *Anaerostipes*, *Faecalicatena*, (171 positive, 1 negative edges; Fig. 7A). However, given the small control sample size (*n* = 9), this network is likely underpowered for reliable edge recovery and may therefore appear spuriously sparse or modular.

The IBS network displayed reduced clustering and modularity, enriched in fermentative fungal keystones from Ascomycota (*Tolypocladium*, *Pseudoanungitea*) and bacterial keystones including *Parabacteroides*, *Acidaminococcus*, and *Christensenellaceae* (569 positive, 259 negative edges; Fig. 7B). These features were consistent with the observed enrichment of fermentative taxa in IBS across domains.

In contrast, the UC network exhibited a fragmented topology with low modularity and prominent fungal keystones (*Trichoderma*, *Tolypocladium*, *Nigrospora*) linked to bacterial taxa such as *TM7x*, *Segatella*, and *Terrisporobacter* (642 positive, 268 negative edges; Fig. 7C). Robustness analysis indicated heightened structural vulnerability (AUC = 0.281; R50 = 0.256), with rapid loss of connectivity following targeted removal of high-betweenness nodes, reflecting dependence on a limited set of bridging taxa. These features were consistent with UC-associated bacterial networks enriched for D-type succinotype configurations.

The CD network displayed intermediate organization, retaining moderate modularity and featuring keystone taxa including fungal *Marasmius*, *Chaetomium*, and *Fusicolla*, the archaeon *Methanobrevibacter*, and bacterial genera such as *Oscillospira*, *Anaerotruncus*, and *Weissella* (474 positive, 280 negative edges; Fig. 7D). CD networks retained > 90% connectivity after approximately 10% random node removal but collapsed following targeted hub removal, reflecting reliance on a small keystone genus.

Comparative robustness analyses highlighted disease-associated fragility (Fig. 7E). The small control cohort (*n* = 9) produced a sparsely connected network (174 nodes, 11% LCC), likely reflecting limited statistical power, whereas disease cohorts (IBS *n* = 29, UC *n* = 31, CD *n* = 30) resulted in larger and denser networks (265–273 nodes, 93–99% LCC). Robustness indices (AUC) were highest in UC (0.28), intermediate in IBS (0.25) and controls (0.24), and lowest in CD (0.22). These patterns suggest that apparent resilience in controls may be influenced by sample size, while disease-associated cohorts reveal distinct network organizations shaped by dysbiotic taxa and altered associations.

Fragmentation dynamics further distinguished disease groups: control networks decreased early (LCC halved at ~ 7% node removal) but plateaued mid-stage, whereas disease networks fragmented later (15–26%) yet collapsed more rapidly (39–43%), with CD showing the longest late-stage plateau (~ 72 steps at ~ 1% LCC). These patterns are consistent with previous ecological network studies in which fragile topologies reflect reduced redundancy and increased susceptibility to perturbation.

Taken together, bacterial and fungal microbiomes across gastrointestinal disorders exhibited distinct disease-associated network configurations, with inflammation status, symptoms, and enterotype/*Blastocystis* carriage modulating observed associations. UC was characterized by loss of butyrate-associated taxa, enrichment of opportunistic fungi, and highly vulnerable network structures, whereas IBS displayed enrichment of fermentative taxa across domains within comparatively more resilient networks. CD exhibited intermediate organization with partial robustness.

## Discussion

In this study, we provide an integrated analysis of bacterial and fungal communities across gastrointestinal disorders, linking microbial composition, diversity, and network properties to host inflammation, symptoms, and ecological covariates. We show that bacterial and fungal microbiomes exhibit distinct yet complementary disease-associated signatures: UC was characterized by loss of butyrate producers, enrichment of opportunistic fungi, and fragile community networks; CD displayed depletion of methanogens and *Clostridia UCG-014*, with intermediate network resilience; and irritable bowel syndrome (IBS) was marked by increased bacterial evenness and enrichment of fermentative fungi. Importantly, we demonstrate that many associations are modified by host inflammation and ecological covariates such as enterotype and *Blastocystis* carriage, underscoring the need to consider both host physiology and microbial ecology in interpreting microbiome-disease relationships. Finally, network fragility in IBD may represent a disease-associated ecological feature and a candidate mechanistic hypothesis linking microbial community organization with inflammatory states. This pattern points to ecosystem instability as a potential mechanism underlying disease pathogenesis.

We identified two *Bacteroides*-dominant enterotypes (Bact1/Bact2) aligned with prior studies linking enterotype stratification to gut ecosystem stability and inflammation [40, 41]. Bact2, characterized by reduced alpha diversity and enrichment of *Escherichia-Shigella* and *Streptococcus*, mirrors enterotypes associated with obesity and IBD in Western cohorts [42, 43]. Bact2 was strongly associated with calprotectin positivity, reinforcing its role as a microbial signature of intestinal inflammation [40]. However, unlike previous reports [40, 41], enterotype distribution did not differ significantly across disease groups, suggesting that enterotypes may reflect microbial community resilience or inflammatory tone rather than specific diagnoses. This observation emphasizes the need to consider additional axes of microbial variation, such as metabolic niches captured by succinotypes, to disentangle disease-associated dysbiosis.

Stratification by succinate-utilizing taxa revealed UC-specific enrichment of D-type (*Dialister*-dominant) communities, which correlated with elevated faecal succinate levels. Succinate accumulation is a known driver of macrophage activation and intestinal inflammation [44], and our observation aligns with prior work showing that *Dialister*-dominant microbiomes are linked to IBD but not IBS, suggesting their association with chronic inflammation or heightened inflammatory risk [45]. The enrichment of *Dialister* and related taxa in the D-type aligns with prior reports linking these organisms to succinate metabolism and inflammatory states. Nevertheless, succinate levels were not directly quantified here, and the proposed functional link should be interpreted as hypothesis-generating.

On the contrary, P-type (*Phaseolaretobacterium*-dominant) communities, enriched in IBS, may promote anti-inflammatory butyrate production through cross-feeding with *Faecalibacterium*, a process supported by in vitro studies demonstrating synergistic butyrogenesis between these taxa [46]. The association of the P-type with taxa such as *Faecalibacterium* and *Roseburia*, which are well-established butyrate producers, suggests a potentially higher butyrate-producing capacity of this microbial configuration. However, butyrate concentrations were not directly measured in this study, and these functional inferences are based on known metabolic capabilities reported in the literature.

The higher prevalence of mixed succinotypes (18%) compared to prior studies [45, 47] highlights the underappreciated complexity of succinate metabolism in non-Western populations, potentially influenced by regional dietary patterns. Network analysis further revealed that mixed-type communities harboured keystone taxa (*Clostridia UCG-014*, *Megasphaera*) bridging mucin degradation and butyrate production pathways, suggesting metabolic flexibility that stabilizes dysbiotic states in UC [48, 49]. These findings extend the framework of succinotype-driven inflammation [45, 47] by elucidating ecological mechanisms that sustain microbial community resilience in IBD.

Gut fungal communities exhibited pronounced disease-associated contrasts, though the mycobiome is heavily influenced by environmental and dietary transients. Fungal communities were dominated by Basidiomycota and Ascomycota, with *Malassezia*, *Saitozyma*, and *Rhodotorula* among the most abundant taxa. UC and CD were enriched in opportunistic fungi, particularly *Candida*, *Saccharomyces*, and *Malassezia*, consistent with prior observations linking these taxa to mucosal inflammation in IBD [2, 50]. Basidiomycota/Ascomycota ratios were higher in UC and CD compared to IBS, reflecting stronger dysbiosis in IBD. For IBS, fungal profiles were less pronounced and highly variable, with modest enrichment of fermentative taxa (*Candida*, *Meyerozyma*, *Wickerhamomyces*) primarily in non-inflammatory samples; these patterns remain exploratory due to limited evidence [51].

Calprotectin-stratified analyses revealed *Didymella* as a candidate UC-specific biomarker, independent of inflammation, highlighting fungal taxa as candidate indicators. These results emphasize that bacterial and fungal diversity are driven by distinct ecological and host factors: bacterial shifts largely reflect enterotype, succinotype, and inflammatory status, whereas fungal patterns are influenced by environmental exposure and host physiology.

*Blastocystis* was detected in 48% of participants, predominantly ST1 and ST3. Neither presence nor subtype was significantly associated with host characteristics or enterotype/succinotype. *Blastocystis* carriage explained 1.4–2.6% of bacterial beta diversity, acting as a secondary ecological covariate. Positive carriers were enriched in butyrate-producing bacteria (*Roseburia*, *Anaerobutyricum*, *Butyribacter*), whereas negatives were enriched in Bacteroides. Subtype-specific differences were exploratory, with ST1 enriched in *Alistipes* and ST3 in *Escherichia-Shigella* and *Klebsiella*, in line with prior studies [52, 53]. These findings support positioning *Blastocystis* as a modulator of community structure rather than a primary disease driver.

Given the small sample size of the control group in this cohort, comparisons involving controls are therefore underpowered. As a result, these taxa should be interpreted as exploratory signals that provide directional insight into disease-associated microbial patterns rather than as validated diagnostic markers. Their primary value lies in hypothesis generation and in informing future studies with larger, independent control cohorts. Notably, key ecological and network-level conclusions in this study are supported by disease-disease contrasts and inflammation stratified analyses, which are less sensitive to control sample size.

Network inference from compositional microbiome data is inherently sensitive to sample size, particularly with respect to individual edge recovery. The small control group (*n* = 9) is therefore most susceptible to underpowered and potentially sparse networks. By contrast, disease cohort (*n* ≈ 30) fall within the sample-size range for which SPIEC-EASI has been benchmarked and is commonly applied in microbiome studies [34]. Accordingly, we interpret inferred graphs as cohort-level conditional dependence structures and emphasize relative, disease-associated differences in global network organization and vulnerability patterns rather than population-level or edge-specific truth.

Cross-domain network analyses revealed marked restructuring of bacterial-fungal associations across GIT disorders. Control networks were highly modular, with keystone taxa including butyrate-producing bacteria (*Anaerostipes*, *Faecalicatena*) and fungi (*Saccharomyces*, *Malassezia*). IBS networks exhibited modest fragility, retaining fermentative fungal taxa (*Candida*, *Meyerozyma*), whereas UC networks were most fragile, with loss of connectivity upon keystone removal and enrichment of opportunistic fungi (*Candida*, *Malassezia*). CD networks showed intermediate robustness, reflecting partial resilience. These observations align with D-type succinotype enrichment in UC and P-type resilience in IBS, integrating community composition with network architecture. Fragile networks in UC underscore ecosystem instability as a potential mechanistic contributor to disease pathogenesis [54]. Fragile networks in UC are consistent with ecosystem instability accompanying disease and inflammation; however, causal direction cannot be inferred in this cross-sectional design and unmeasured exposures such as diet may contribute to observed network restructuring.

Collectively, our findings highlight that microbial signatures in GI disorders reflect the intersection of host inflammation, microbial ecology, and transient colonizers. UC-associated dysbiosis is characterized by succinate-driven D-type bacterial enrichment and opportunistic fungal expansion, whereas IBS is associated with metabolically flexible P-type bacteria and fermentative fungi. CD displays intermediate patterns, with depletion of methanogens and selective fungal enrichments. These insights suggest that multi-kingdom interactions shape disease-associated microbial landscapes. Limitations include modest cohort size, especially controls, cross-sectional design, and the transient nature of environmental fungi complicating mycobiome interpretation. An additional limitation is the absence of detailed dietary intake data; therefore, future studies in this population should incorporate standardized dietary assessment to disentangle disease effects from diet-linked ecological shifts. There are no direct metabolite measurements (e.g., faecal succinate or short-chain fatty acids), which precludes direct functional validation of the inferred metabolic potential suggested by taxonomic composition. Future longitudinal, multi-omics, and mechanistic studies (e.g., gnotobiotic models) are warranted to validate causality and inform intervention strategies targeting succinate metabolism, microbial networks, and fungal biomarkers. Longitudinal sampling and experimental perturbation studies will be required to establish temporal ordering and causality between microbial network stability and disease activity.

Furthermore, SPIEC-EASI network inference does not explicitly account for host or environmental covariates unless they are directly modelled. Accordingly, some inferred microbial associations may reflect shared responses to unmeasured factors such as diet, medication or age rather than direct ecological interactions. This limitation is common to graphical-model-based microbiome network approaches and has been widely discussed in the literature. In this study, we therefore emphasize disease-associated differences in global network organization and vulnerability patterns rather than causal interpretation of individual edges. Future work incorporating covariate-aware methods (e.g., FlashWeave) or longitudinal designs will be required to disentangle disease effects from environmentally driven microbial restructuring.

## Conclusion

This study characterized the structural and inferred functional features of the gut microbiome in patients with irritable bowel syndrome (IBS) and inflammatory bowel disease (IBD) by integrating bacterial and fungal community profiling in a Saudi Arabian cohort. By combining gut-type classification, succinate-type stratification, protozoan carriage status, and cross-domain network analysis, we demonstrate that disease status and host-associated factors are jointly associated with microbial community organization.

IBD, particularly ulcerative colitis, was associated with enrichment of succinate-metabolizing (D-type) bacterial configurations, fungal dysbiosis, and increased fragility of bacterial–fungal interaction networks, whereas IBS was characterized by higher bacterial evenness, enrichment of fermentative fungal taxa, and comparatively more resilient microbial networks. Two Bacteroides-dominated gut types (Bact1 and Bact2) were identified, with Bact2 showing significant associations with inflammatory markers. In addition, *Blastocystis* carriage was linked to enrichment of taxa with established butyrate-producing capacity.

Together, these findings support a multidimensional, multi-kingdom view of gut microbial ecology and highlight disease-associated differences in microbial community structure and network organization in a non-Western population. While causal relationships cannot be inferred from this cross-sectional design, the proposed framework of gut type-succinate type-network resilience, provides an ecological context for understanding microbiome variation in gastrointestinal disease and offers a foundation for future longitudinal and functional studies aimed at microbiome-informed diagnostics and interventions.

## Supplementary Information

Below is the link to the electronic supplementary material.

Supplementary Material 1

## Data Availability

Raw sequencing data are deposited in the National Center for Biotechnology Information (NCBI) Sequence Read Archive (SRA) under BioProject PRJNA1335553. Additional datasets and materials are available upon reasonable request from the corresponding author.
