# Supplementary material for: Bacterial and fungal biomarkers in irritable bowel syndrome (IBS) and inflammatory bowel disease (IBD): trans-kingdom interactions, Blastocystis carriage, and enterotype–succinotype stratification
Source: Gut Pathog. 2026 Apr 11;18:34. doi: 10.1186/s13099-026-00819-3 (PMC13091276; doi:10.1186/s13099-026-00819-3)
Supplement: Supplementary file 1 — Supplementary Material 1 [file 13099_2026_819_MOESM1_ESM.docx]

**Supplementary tables and figures**

**Supplementary tables**

Supplementary table 1. Clinical characteristics and GI symptoms across different groups.

| **Characteristic** | **Controls** | **N** | **IBS** | **N** | **UC** | **N** | **CD** | **N** |
| --- | --- | --- | --- | --- | --- | --- | --- | --- |
| Age, years (median (min, max)) | 41 (14, 60)^*^ | 9 | 36 (7, 85) ^**^ | 29 | 31 (10, 62) | 31 | 24 (9, 60)^*/**^ | 30 |
| Age group (7-25, %) | 11 |  | 24 |  | 32 |  | 53 |  |
| 25-38 (%) | 33 |  | 34 |  | 32 |  | 33 |  |
| 38-85 (%) | 56 |  | 41 |  | 35 |  | 13 |  |
| Sex (Female, %) | 56 | 9 | 52 | 29 | 39 | 31 | 50 | 30 |
| Abdominal symptoms (Yes, %) | 44 | 9 | 55^*^ | 29 | 29 | 31 | 17^*^ | 30 |
| Constipation (Yes, %) | 11 | 9 | 6.9 |  | - | 31 | - | 30 |
| Anorexia (Yes, %) | 22 | 9 | 21 | 29 | 3.2 | 31 | 3.3 | 30 |
| Nausea (Yes, %) | 33 | 9 | 38^*/**^ | 29 | 13^*^ | 31 | 6.7^**^ | 30 |
| Vomiting (Yes, %) | 44^*^ | 9 | 34^**^ | 29 | 16 | 31 | 6.7^*/**^ | 30 |
| Dyspepsia heartburn (Yes, %) | 33^*^ | 9 | 28^**^ | 29 | 9.7 | 31 | 3.3^*/**^ | 30 |
| Rectal bleeding (Yes, %) | 11 | 9 | 6.9 | 29 | 6.5 | 31 | 10 | 30 |
| Stool blood (Yes, %) | 11 | 9 | 6.9 | 29 | 19 | 31 | 6.7 | 30 |
| Diarrhoea (Yes, %) | 22 | 9 | 41 | 29 | 35 | 31 | 27 | 30 |
| Abdominal pain (Yes, %) | 44 | 9 | 79^*/**^ | 29 | 42^*^ | 31 | 43^**^ | 30 |
| Calprotectin (Positive, %) | 100 | 2 | 33 | 6 | 72 | 29 | 62 | 29 |
| *Blastocystis* (Positive, molecular, %) | 78 | 9 | 55 | 29 | 52 | 31 | 47 | 30 |
| *Blastocystis* subtypes (ST1, %) | 67 | 9 | 41 | 29 | 32 | 31 | 33 | 30 |
| ST3 | 11 |  | 14 |  | 9.7 |  | 10 |  |
| ST7 | 0 |  | - |  | 3.2 |  | - |  |

Groups with similar superscripts (^*^ and ^**^) indicate statistically significant differences between the respective groups for each characteristic. Pairwise Wilcoxon test was used to test the difference in age between the controls, and inflammatory bowel syndrome and disease (UC and CD) groups (*P* < 0.05). Fisher's exact test was used to test the difference between IBS; Inflammatory bowel disease, UC; Ulcerative colitis, CD; Crohn’s disease, Molecular detection was done using cPCR; conventional polymerase chain reaction and RTPCR; real-time polymerase chain reaction. *Blastocystis* was classified as PCR-positive if detected by either cPCR, RT-PCR, or both, and PCR-negative only if negative by both methods.

Supplementary table 2. Differential abundance of bacterial and fungal genera between different succinotype groups using Wilcoxon signed-rank test

| Genus | D-type (mean (%)) | P-type (mean (%)) | Mixed (mean (%)) | Group-1 | Group-2 | statistic | BH P value |
| --- | --- | --- | --- | --- | --- | --- | --- |
| *[Eubacterium]_coprostanoligenes_group_Incertae_Sedis* | 1.69 | 0.59 | 1.68 | P-type | Mixed | 145 | 0.03 |
| *[Eubacterium]_eligens_group* | 0.19 | 0.53 | 1.01 | D-type | Mixed | 219 | 0.03 |
| *[Eubacterium]_ruminantium_group* | 0.19 | 0.17 | 0.39 | D-type | Mixed | 232 | 0.02 |
| *[Eubacterium]_ruminantium_group* | 0.19 | 0.17 | 0.39 | P-type | Mixed | 145 | 0.02 |
| *Bacteroides* | 17.5 | 26.1 | 12.3 | P-type | Mixed | 386 | 0.02 |
| *Bacteroides* | 17.5 | 26.1 | 12.3 | D-type | P-type | 405 | 0.03 |
| *Bilophila* | 0.11 | 0.47 | 0.25 | P-type | Mixed | 369 | 0.03 |
| *Bilophila* | 0.11 | 0.47 | 0.25 | D-type | P-type | 279 | 0.00033 |
| *Butyribacter* | 0.04 | 0.09 | 0.19 | D-type | Mixed | 229 | 0.02 |
| *Christensenellaceae_R-7_group* | 1.43 | 0.93 | 0.86 | P-type | Mixed | 146 | 0.04 |
| *Clostridia_UCG-014_Incertae_Sedis* | 0.55 | 0.19 | 1.24 | D-type | Mixed | 198 | 0.01 |
| *Clostridia_UCG-014_Incertae_Sedis* | 0.55 | 0.19 | 1.24 | P-type | Mixed | 81 | 0.0002 |
| *Clostridioides* | 0.18 | 0.07 | 0.36 | D-type | Mixed | 282 | 0.04 |
| *Coprobacter* | 0.02 | 0.01 | 0.02 | D-type | Mixed | 182 | 0.001 |
| *Coprobacter* | 0.02 | 0.01 | 0.02 | P-type | Mixed | 131 | 0.002 |
| *Dialister* | 5.32 | 0.01 | 2.26 | D-type | Mixed | 536 | 0.01 |
| *Dialister* | 5.32 | 0.01 | 2.26 | P-type | Mixed | 23 | 0 |
| *Dialister* | 5.32 | 0.01 | 2.26 | D-type | P-type | 1192 | 0 |
| *DTU089* | 0.04 | 0.01 | 0 | D-type | P-type | 846.5 | 0.01 |
| *Enterocloster* | 1.24 | 1.59 | 0.64 | P-type | Mixed | 366 | 0.03 |
| *Enterocloster* | 1.24 | 1.59 | 0.64 | D-type | P-type | 388 | 0.03 |
| *Eubacterium* | 0 | 0 | 0 | D-type | Mixed | 315 | 0.01 |
| *Eubacterium* | 0 | 0 | 0 | D-type | P-type | 483 | 0.01 |
| *Faecalicatena* | 0.02 | 0.02 | 0 | P-type | Mixed | 377 | 0.03 |
| *Faecalicatena* | 0.02 | 0.02 | 0 | D-type | P-type | 426 | 0.05 |
| *Haemophilus* | 0.71 | 0.33 | 1.45 | P-type | Mixed | 131.5 | 0.01 |
| *Hungatella* | 0.05 | 1.31 | 1.11 | D-type | P-type | 367 | 0.01 |
| *Lachnoclostridium* | 0.16 | 0.45 | 0.02 | P-type | Mixed | 376 | 0.02 |
| *Lachnoclostridium* | 0.16 | 0.45 | 0.02 | D-type | P-type | 359 | 0.01 |
| *Methylorubrum* | 0.01 | 0 | 0.01 | D-type | Mixed | 276 | 0.03 |
| *Methylorubrum* | 0.01 | 0 | 0.01 | P-type | Mixed | 193 | 0.04 |
| *NK4A214_group* | 0.55 | 0.34 | 0.85 | P-type | Mixed | 146 | 0.03 |
| *Phascolarctobacterium* | 0.03 | 5.55 | 1.12 | D-type | Mixed | 42 | 0 |
| *Phascolarctobacterium* | 0.03 | 5.55 | 1.12 | P-type | Mixed | 405 | 0.001 |
| *Phascolarctobacterium* | 0.03 | 5.55 | 1.12 | D-type | P-type | 33 | 0 |
| *Streptomyces* | 0 | 0.01 | 0.04 | D-type | Mixed | 282 | 0.03 |
| *Streptomyces* | 0 | 0.01 | 0.04 | P-type | Mixed | 192 | 0.03 |
| *Turicimonas* | 0 | 0.05 | 0 | D-type | Mixed | 301 | 0.03 |
| *UCG-005* | 0.5 | 0.2 | 0.8 | P-type | Mixed | 149 | 0.04 |
| *Veillonella* | 2.4 | 0.5 | 1.27 | D-type | Mixed | 215 | 0.01 |
| *Veillonella* | 2.4 | 0.5 | 1.27 | P-type | Mixed | 124 | 0.01 |

Supplementary table 3 Differential abundance of bacterial and fungal genera between *Blastocystis* carriers and non-carriers, and across *Blastocystis* subtypes Wilcoxon signed-rank test

| **Bacterial differential abundance** | **Blastocystis (Positive/Negative)** | | | | | |
| --- | --- | --- | --- | --- | --- | --- |
| **Genus** | **Positive**  **(mean %)** | **Negative**  **(mean %)** | **Group-1** | **Group-2** | **statistic** | **BH P value** |
| *Phascolarctobacterium* | 2.49 | 1.43 | 48 | 50 | 1481 | 0.0442 |
| *Mitsuokella* | 0.05 | 0.08 | 48 | 50 | 1347 | 0.0475 |
| *Anaerococcus* | 5.18E-05 | 0.004 | 48 | 50 | 960.5 | 0.00285 |
| *Peptostreptococcus* | 0.0007 | 0.014584806 | 48 | 50 | 985 | 0.0402 |
| *Paludicola* | 0.00617 | 0.00598 | 48 | 50 | 1455.5 | 0.0101 |
| *unclassified_Oscillospiraceae* | 0.079 | 0.048 | 48 | 50 | 1525 | 0.0191 |
| *Anaerobutyricum* | 0.35 | 0.075 | 48 | 50 | 1613 | 0.0033 |
| *Butyribacter* | 0.11 | 0.058 | 48 | 50 | 1452.5 | 0.0413 |
| *Fusicatenibacter* | 0.22 | 0.31 | 48 | 50 | 1480 | 0.0466 |
| *Roseburia* | 1.47 | 0.75 | 48 | 50 | 1531 | 0.0188 |
| *Neisseria* | 0 | 0.013 | 48 | 50 | 1080 | 0.0259 |
| *Leyella* | 0.12 | 0.98 | 48 | 50 | 973 | 0.0302 |
| *Bacteroides* | 12.81 | 24.41 | 48 | 50 | 847 | 0.0122 |
|  | **Subtype (ST1/ST3)** | | | | | |
| **Genus** | **Positive (mean %)** | **Negative (mean %)** | **Group-1** | **Group-2** | **statistic** | **BH P value** |
| *Anaeroglobus* | 3.15E-05 | 0.001 | 38 | 11 | 157 | 0.00936 |
| *Fusobacterium* | 0.97 | 0.68 | 38 | 11 | 130.5 | 0.0383 |
| *Terrisporobacter* | 0.009 | 0 | 38 | 11 | 291.5 | 0.0161 |
| *Klebsiella* | 0.51 | 0.47 | 38 | 11 | 120 | 0.0303 |
| *Escherichia-Shigella* | 2.92 | 6.35 | 38 | 11 | 105 | 0.0131 |
| *Agathobacter* | 2.57 | 0.31 | 38 | 11 | 297.5 | 0.0348 |
| *Eikenella* | 6.49E-05 | 0.001 | 38 | 11 | 160 | 0.027 |
| *Alistipes* | 4.01 | 0.68 | 38 | 11 | 323 | 0.00653 |
| **Fungal differential abundance** | **Blastocystis (Positive/Negative)** | | | | | |
| **Genus** | **Positive (mean %)** | **Negative (mean %)** | **Group-1** | **Group-2** | **statistic** | **BH P value** |
| *Nigrospora* | 2.67E-05 | 0.000157171 | 47 | 50 | 938 | 0.04 |
| *Capronia* | 6.92E-05 | 5.48E-05 | 47 | 50 | 1403 | 0.04 |
| *Tympanidaceae_gen_Incertae_sedis* | 0.00041 | 0.00018 | 47 | 50 | 1461 | 0.03 |
| *Naganishia* | 0.00073 | 8.56E-05 | 47 | 50 | 1353 | 0.02 |
| *Alternaria* | 0.00135 | 0.0036 | 47 | 50 | 873 | 0.03 |
| *Lunulospora* | 0.00015 | 4.29E-05 | 47 | 50 | 1549 | 0.002 |
|  | **Subtype (ST1/ST3)** | | | | | |
| **Genus** | **ST1 (mean %)** | **ST3 (mean %)** | **Group-1** | **Group-2** | **statistic** | **BH P value** |
| *Marquandomyces* | 5.94E-06 | 3.07E-05 | 37 | 11 | 133 | 0.02 |
| *Zygosporium* | 0.00015 | 1.07E-05 | 37 | 11 | 297 | 0.01 |
| *unclassified_Sclerotiniaceae* | 0.002747514 | 0.00083 | 37 | 11 | 306.5 | 0.01 |
| *Sporormiaceae_gen_Incertae_sedis* | 0.00024 | 3.05E-06 | 37 | 11 | 296 | 0.01 |
| *Didymella* | 7.14E-06 | 0.0029 | 37 | 11 | 130 | 0.01 |

**Supplementary figures**

| A) | B) |
| --- | --- |
| 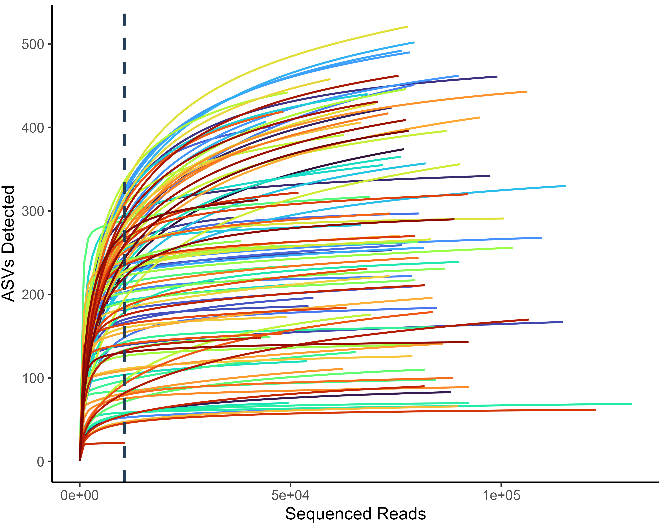 | 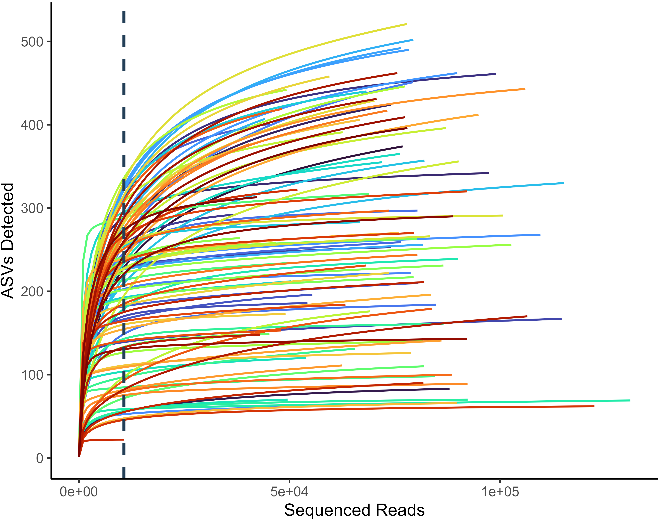 |

**Figure 1.** Rarefaction curve showing the relationship between sequencing depth and detected amplicon sequence variants (ASVs) for each sample. A) 16S rRNA sequence, and B) ITS sequence. The dashed line indicates the minimum sequencing depth of 25,261 reads across samples.


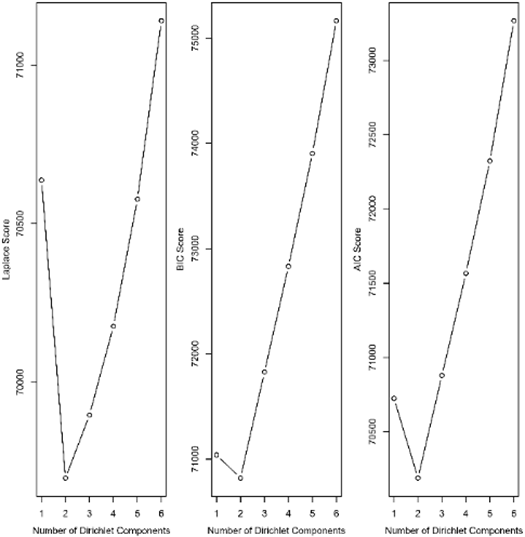


**Figure 2.** Enterotype model fit assessed using the Laplace score, Bayesian Information Criterion (BIC), and Akaike Information Criterion (AIC).
